# The interference of digital radiographic image acquisition and processing protocols in the diagnosis of incipient enamel carious lesions

**DOI:** 10.4317/jced.60990

**Published:** 2024-02-01

**Authors:** Patricia-Fernandes-Avila Ribeiro, Lina-Naomi Hashizume, Luis-Ernesto Arriola-Guillén, Kristian Madeira, Mariana-Boessio Vizzotto, Heraldo-Luís-Dias da Silveira

**Affiliations:** 1 Ph.D.(c) Dental School, Federal University of Rio Grande do Sul (UFRGS), Porto Alegre, Rio Grande do Sul, Brazil; 2 DDS, MS, PhD. Associate Professor of the Division of Caries and Microbiology, Dental School, Federal University of Rio Grande do Sul (UFRGS), Porto Alegre, Rio Grande do Sul, Brazil; 3 Ph.D. and Associate Professor of the Division of Orthodontics and Division of Oral and Maxillofacial Radiology, School of Dentistry, Universidad Científica del Sur, Lima, Perú; 4 Ph.D. and Professor of the Postgraduate Program in Health Sciences, University of Southern Santa Catarina, Criciúma, Santa Catarina, Brazil; 5 DDS, MS, PhD. Associate Professor of the Department of Oral Surgery and Orthopedics, Division of Dental Radiology, Dental School, Federal University of Rio Grande do Sul (UFRGS), Porto Alegre, Rio Grande do Sul, Brazil; 6 DDS, MS, PhD. Associate Professor of the Department of Oral Surgery and Orthopedics, Division of Dental Radiology, Dental School, Federal University of Rio Grande do Sul (UFRGS), Porto Alegre, Rio Grande do Sul, Brazil

## Abstract

**Background:**

This study aims to evaluate the diagnosis of proximal carious lesions through different parameters of execution and visualization of the images.

**Material and Methods:**

Proximal carious lesions were artificially induced in human teeth three different times (10, 20, and 30 hours) by in vitro demineralization. The teeth were radiographed with the Dürr digital system using three exposure times (0.10, 0.16, and 0.20s) and three different resolutions in the scanning of the phosphor plates (20-, 25- and 40-line pairs). After acquiring the images, they were submitted to VistaScan Fine, Caries 1, and Caries 2 software filters. Three radiologists analyzed the images in JPEG format.

**Results:**

The Pearson’s chi-square test showed an association between longer demineralization time and the presence of injury according to the professional’s classification. There was no statistically significant association among the other parameters, exposure time, resolution, and use of filters and the presence of a lesion. All parameters showed a greater sensitivity than specificity. The exposure time of 0.2s showed better accuracy, the resolutions 25 and 40lp showed equal results and better than 20lp and the Fine filter obtained better values than the Caries 1 and 2 filters.

**Conclusions:**

Despite the variation of parameters, exposure time, resolution, and use of filters, there were no statistically significant differences. For better diagnostic accuracy, it is recommended to use 0.2s of exposure time and a Fine filter, and scan the phosphor plates with 25lp.

** Key words:**Orbital fracture, Etiology, Orbital trauma, Road traffic accidents, Trauma.

## Introduction

Caries remains one of the most prevalent diseases in the world’s population despite major advances in prevention (1). Proximal carious lesions are often diagnosed by the association of clinical and radiographic examinations. The use of interproximal radiography as a complement to the clinical examination allows greater sensitivity in the detection of proximal and occlusal carious lesions as well as a better estimate of the depth of the lesion than visual inspection alone (2).

Current digital radiographic systems allow performing examinations with the use of low-dose receivers (2). Some studies show that image receptors, such as phosphor storage plates (PSP), are more frequently used due to their smaller thickness compared to direct image receptors, being wireless and having a certain degree of flexibility, very similar to traditional radiographic film (3-6). Among the image formation properties of indirect receptors, spatial resolution is defined as the ability to distinguish a radiographic image with precision and detail, measured using line pairs per millimeter (lp/mm) (7).

Among other technological innovations, the tools that digital system software offers for image manipulation, such as brightness, contrast, use of specific filters, and zoom, are of note. These tools can improve or help the radiographic interpretation of several pathologies, including carious lesions. However, if applied improperly, they can degrade the image and impair the diagnostic capacity (8-10). Several studies have shown that only a few filters in the software of digital systems can help detect caries lesions and that their effect on accuracy can vary between systems and between observers in the same system (11).

To improve the diagnostic accuracy of caries lesions, different image processing techniques have been evaluated, including brightness, noise reduction or pseudo coloration. In summary, the use of different filters has improved the ability to diagnose caries lesions. However, none previous studies evaluated the use of these three different filters (VistaScan digital system, Caries 1, Caries 2) or compared their sensitivity and precision values, data that can be very useful for clinical activity. Thus, considering the use of available digital systems and software tools constitutes a challenge for the early detection and diagnosis of carious lesions, therefore the objective of this study was to evaluate the diagnosis of proximal carious lesions using different execution parameters and image display.

## Material and Methods

An *in vitro* experimental study was carried out following approval from the Ethics Committee of the Faculty of Dentistry of the Federal University of Rio Grande do Sul, with protocol number 4,536,445. Unerupted third molar teeth were selected for the study in order to avoid interference of the post-eruptive maturation process with the demineralization process.

Calculation of the minimum sample size was based on previous studies (3,10,12,13). The calculation was performed using the Bioestat software, version 5.0, resulting in 52 samples (teeth), divided into four groups of 13 samples each, one control and three experimental. One more element was added to each group to ensure a safety margin in the procedures, totaling 56 teeth.

The crowns were waterproofed with resistant acid varnish, except for areas of 5 mm² located on the interproximal surfaces, protected with adhesives glued on the proximal faces, and were submitted to the demineralization process. The teeth were numbered and randomly divided into four groups (three experimental and one control) with 14 specimens (teeth) each using the Excel program (Microsoft Office Professional Plus 2019).

The demineralization methodology employed followed the model by El-Ela, Farid and Mostafa (2016) (13) using 5% formic acid. The specimens of the experimental groups were kept individually submerged in 8 ml of solution at 37ºC for 10, 20, and 30 hours. Specimens from the control group were kept in individual plastic containers with cotton swabs soaked in distilled water.

Afterwards, a new randomization was performed to select teeth with different demineralization times, and these were grouped into four, forming blocks to be radiographed. The teeth were placed in an endodontic dummy up to 2 mm from the cement-enamel junction level. Additionally, premolars not included in the study were placed in the dummy to complete the group of teeth and better simulate a real situation of interproximal radiography. The teeth were mounted so that the most prominent parts of the proximal faces were in contact and at the same height, to simulate the normal anatomical position. The blocks were numbered and recorded with the time of demineralization of each tooth.

**Figure 1:**
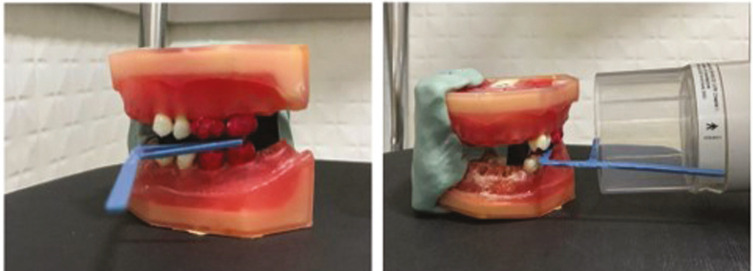
Positioning of the equipment for performing the interproximal radiographic technique.

To simulate interproximal radiography, the teeth were positioned on the dummy at the locations of teeth 26, 27, 37, and 36. An additional silicone guide was made to ensure that all radiographs were taken in the same position. The interproximal positioner was placed between the teeth and the locating cylinder at an 8º vertical angle as shown in Figure 1.

The radiographs were taken with a KaVo FOCUS periapical device (Instrumentarium, Tuusula, Finland) operating at 70Kvp, 7mA, and a focus-film distance of 20 cm. A size 2 phosphor plate of the VistaScan system was used with 3 different exposure times - 0.10s, 0.16,s and 0.20s - to identify possible changes in image quality with the radiation dose recommended by the manufacturer, with an underdose and with overdose of X-radiation following the methodology by Galvão *et al*. (2019) (14).

**Figure 2:**
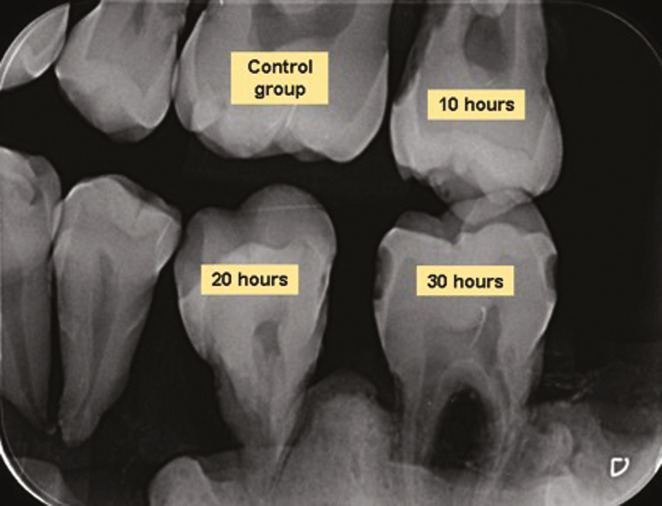
Interproximal X-ray of sampled teeth.

The phosphor plates were scanned in the appropriate equipment using the DBSWIN software (Dürr Dental, Beitigheim-Bissingen, Germany), using 20, 25, and 40 lp. Fine, Caries 1, and Caries 2 filters were applied to each image, saved in JPEG files, and exported separately (Fig. 2).

Combinations of exposure time, resolution, and filters were made for each group of teeth. Thus, each group of teeth was radiographed 27 times according to predetermined combinations.

The images were made available to radiology specialists through Google Docs (Google, INC. Mountais View, California, USA).

Three experienced and calibrated dental surgeons specializing in radiology, who were not part of the research team, calibrated the images prior to evaluation. This analysis was carried out in the work environment of each specialist using the conditions that each evaluator considered the most appropriate according to the study by Lima *et al*. (2020) (15) which tested different viewing conditions, such as different monitors, horizontal angulation variation and different ambient lighting, for the detection of proximal caries in digital radiographic images and concluded that the viewing conditions did not influence the diagnosis. For calibration, 10% of the sample was used, and reassessed at an interval of 15 days, and intra and inter examiner reproducibility was determined using the Kappa index. The observers were experienced radiologists and were free to analyze the images with no minimum number per period.

**Figure 3:**
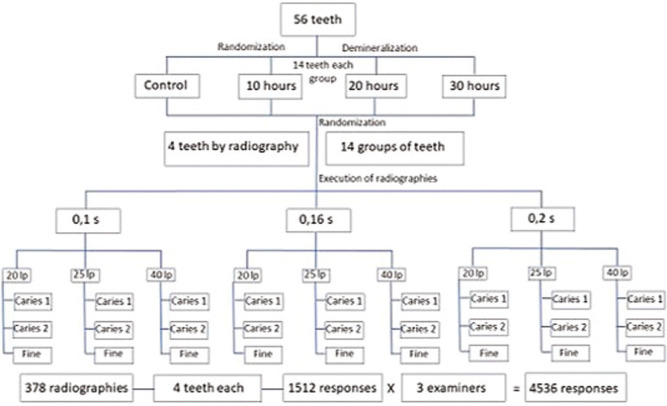
Flowchart of the methodological sequence.

The simulated artificial carious lesion was characterized by a radiolucent area in the proximal enamel. The previously coded radiographs were presented in random order using the Excel program (Microsoft Office Professional Plus 2019), and individually accessed by the observers, who reported the presence or absence of a carious lesion in each tooth using a scale ranging from definitely absent, probably absent, doubtful, probably present and definitely present. After the examiners’ analysis, the answers were dichotomized into the presence and absence of injury, and doubtful answers were classified as the absent group (Fig. 3).

The data collected were organized in a Microsoft Excel spreadsheet and imported into the IBM Statistical Package for the Social Sciences (SPSS) software, version 23.0. All inferential statistical analyses were performed with a significance level of 5% (α = 0.05) and a 95% confidence interval (CI) (15). Pearson’s chi-square test of association was applied to verify whether the correct or incorrect diagnoses made by the three evaluators were associated with the different types of protocols used, the time of X-ray exposure, the three different resolutions, and the three filters. Diagnostic tests were applied to verify sensitivity, specificity, positive predictive value, negative predictive value, and accuracy in comparison with the gold standard (intact teeth and non-intact teeth).

## Results

In the calibration phase, 37 radiographic images were analyzed by each of the three professionals in the first evaluation. Fifteen days later, the second evaluation of the same 37 radiographs was carried out to calculate the Kappa index and verify the degree of agreement between the evaluators and between their own diagnoses made at the two different times. In the intra-observer agreement analysis, evaluators 1, 2, and 3 presented Kappa values of 0.604 (good), 0.947 (very good), and 0.721 (good), respectively, and significant (*p-value* < 0.001), according to Altman (1991) (16). In the inter-observer analysis, evaluator 1 showed moderate agreement with evaluators 2 and 3 (Kappa = 0.523 and 0.510) in the first evaluation and good (0.656) and moderate agreement (0.599) in the second evaluation time (after 15 days), respectively. Evaluators 2 and 3 showed good agreement (0.748 and 0.699) in both evaluations. All Kappa indices were statistically significant, with a *p-value* < 0.001.

The total sample consisted of 56 teeth submitted to 27 radiographs with different combinations of exposure time (0.1 s, 0.16 s and 0.2 s), resolutions (20 lp, 25 lp and 40 lp, and filters (Caries 1, Caries 2 and Fine), resulting in 378 radiographs in which four teeth were evaluated, generating 1512 responses per evaluator, totaling 4536 responses regarding the presence (n = 3294) or absence (n = 1242) of injury.

In regard to the demineralization time, the control group showed the presence of injury in 177 (5.4%) responses, 10 hours in 993 (30.1%) responses, 20 hours in 1009 (30.6%) responses, and 30 hours in 1115 (33.8%) responses. The absence of injury was indicated in the control groups in 957 (77.1%), 10 hours in 141 (11.4%), 20 hours in 125 (10.1%), and 30 hours in 19 (1.5%). The Pearson’s chi-square test showed an association between longer demineralization time and the presence of lesions according to the professional’s classification; that is, the longer the demineralization time, the greater the number of diagnosed lesions (χ^2 = 2510.5; *p* < 0.001) (Table 1).

**Table 1:**
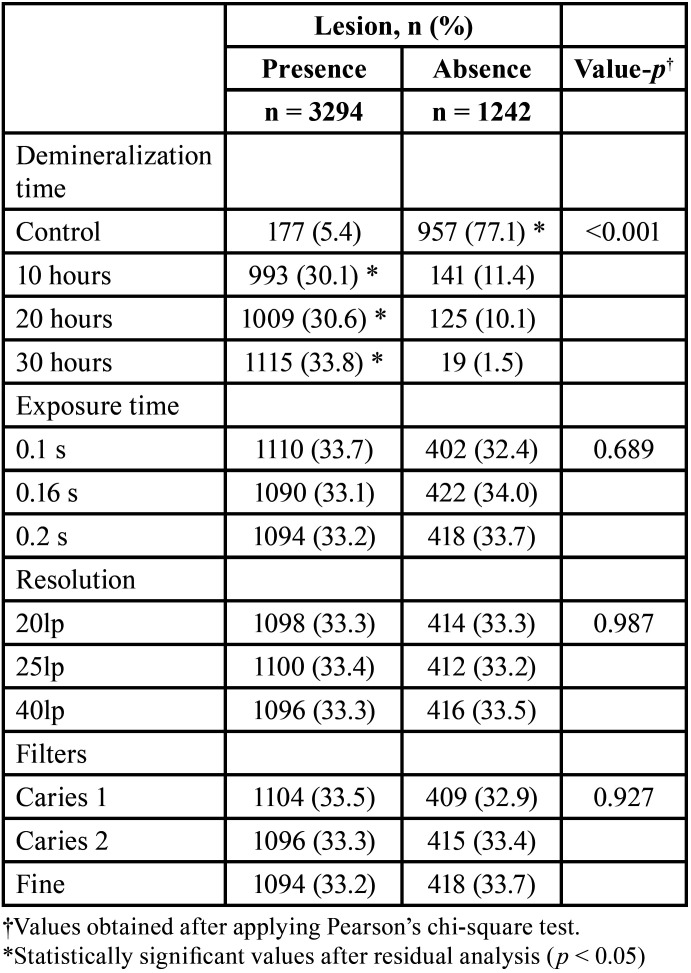
Assessment of the presence or absence of lesions at different times of demineralization, exposure times, resolutions, and filters.

Regarding exposure time, the presence of injury was indicated in the group of 0.1 s in 1110 (33.7%) responses, 0.16 s in 1090 (33.1%) responses and 0.2 s in 1094 (33 .2%) responses. The absence of injury was indicated in the group of 0.1 s in 402 (32.4%) responses, 0.16 s in 422 (34.0%) responses and 0.2 s in 418 (33.7%) responses. The Pearson’s chi-square test showed no association between exposure time and lesion identification (χ^2 = 0.745; gl = 2; *p* = 0.689) (Table 1).

In relation to resolution, the presence of injury was observed in the group of 20lp in 1098 (33.3%) responses, 25lp in 1100 (33.4%) responses and 40lp in 1096 (33.3%) responses. The absence of injury was indicated in the group of 20lp in 414 (33.3%) responses, 25lp in 412 (33.2%) responses and 40lp in 416 (33.5%) responses. The Pearson’s chi-square test showed no association between resolution and lesion identification (χ^2 = 0.27; gl = 2; *p* = 0.987) (Table 1).

Regarding the use of filters, the presence of lesions was observed in the Caries 1 group in 1104 (33.5%) responses, Caries 2 in 1096 (33.3%) and Fine in 1094 (33.2%) responses. The absence of lesions was indicated in the Caries 1 group in 409 (32.9%) responses, Caries 2 in 415 (33.4%) and Fine in 418 (33.7%) responses. The Pearson’s chi-square test showed no association between the use of filters and lesion identification (χ^2 = 0.151; gl = 2; *p* = 0.927) (Table 1).

The sensitivity and specificity and positive predictive value (PPV), negative predictive value (NPV) and accuracy were calculated from the responses of the evaluators, considering intact teeth (not subjected to demineralization) and non-intact teeth as the gold standard (subjected to demineralization).

Considering all the answers obtained (n = 4536), the sensitivity of the evaluators to differentiate between teeth with and without lesions was 91.6% (95% CI 90.7% - 92.6%), the specificity was 84.4% (95% CI 82.3% - 86.5%), the PPV was 94.6% (95% CI 93.9% - 95.4%), the NPV was 77.1% (95% CI 74.7% - 79.4%) and the accuracy was 89.8% (95% CI 88.9% - 90.7%) (Table 2).

**Table 2:**
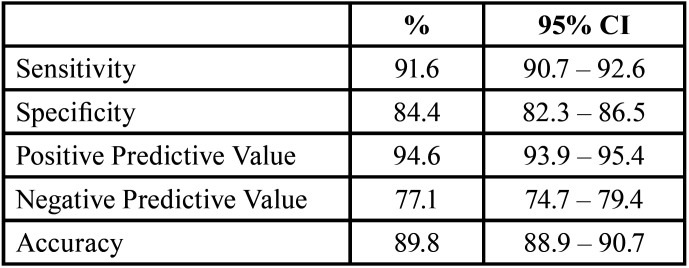
General properties (n = 4536) of the diagnostic test according to the examiners’ assessment compared to the gold standard (intact teeth; non-intact teeth).

Table 3 presents the values of the diagnostic tests for each parameter analyzed in the study, exposure time, resolution and filter (Table 3). Properties according to exposure time, resolution and filters (n = 1512) of the diagnostic test of the examiners’ evaluation compared to the gold standard (intact teeth; non-intact teeth).

**Table 3:**
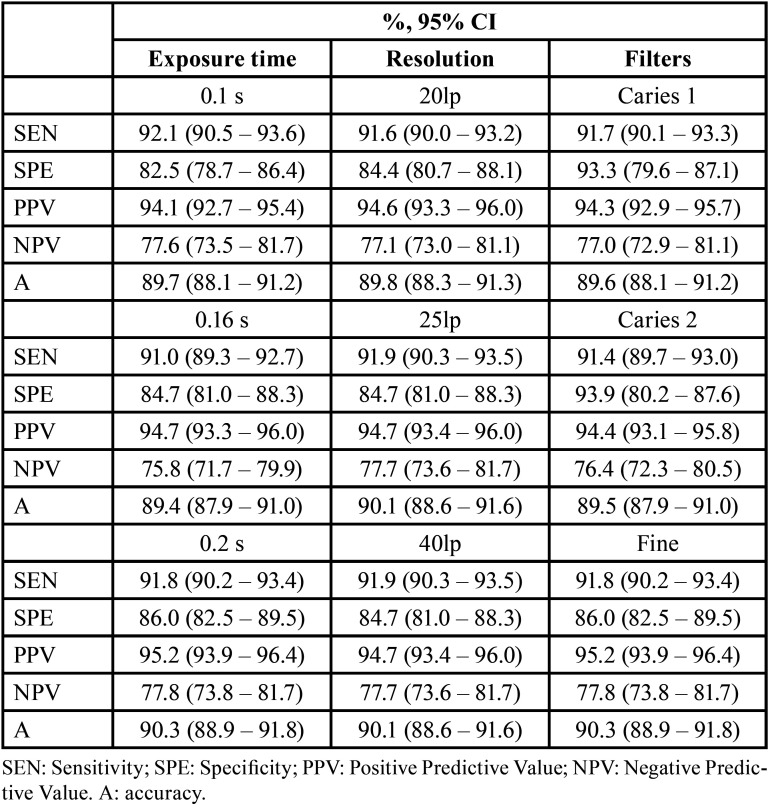
Values of the properties of the diagnostic tests for each parameter analyzed.

## Discussion

The diagnosis of caries disease is made far beyond the presence or absence of a cavity. The detection of proximal lesions in posterior teeth is challenging and the inadequacy of visual and tactile clinical methods is the reason why the use of ionizing radiation to perform interproximal radiographs is recommended (1).

The gold standard to differentiate teeth with or without injury is microscopic analysis, an invasive procedure that can lead to tooth loss (18). In this sense, it is extremely important to know the accuracy of non-invasive diagnostic tests through their sensitivity and specificity and indicators resulting from these, such as positive and negative predictive values. Sensitivity, specificity, and accuracy, in general, verify the ability of a test to differentiate disease from non-disease. Predictive values indicate the probability that the tests are correct for the presence or absence of disease (injury) (19). The results of these tests depend on the evaluation of professionals. In the present study, the evaluators selected, after intra- and inter-examiner reliability tests, showed good or very good agreement, according to Altman (1991) (16).

Simulated carious lesions with different demineralization times showed a significant association with the number of lesions diagnosed by radiologists. The longer the demineralization time, the greater the number of diagnosed lesions, corroborating the data reported by El-Ela, Farid and Mostafa (2016) (13), which showed a strong positive correlation between the duration of demineralization and the histological grading of caries on the surfaces dental.

In relation to the time of radiation exposure, the accuracy achieved with a time of 0.2 s was slightly better than with times of 0.1 and 0.16 s. At all times, the sensitivity was greater than the specificity, showing that diagnosis is likely more correct with the presence rather than the absence of a lesion. Galvão *et al*. (2019) (14) analyzed the influence of high-density radiographic material on automatic exposure compensation in digital systems using different exposure times and found that automatic compensation is influenced by the presence of high-density material and that at different exposure times in different systems Fingerprints can cause increased gray values in dental tissues. Although the influence of the material was not evaluated, the importance of digital imaging diagnostic studies under different variables should be highlighted.

Regarding spatial resolution, which is demonstrated in pairs of lines, several indirect digital systems allow its variation during the scanning of phosphor plates, which could influence the accuracy of the diagnosis. The present study evaluated three resolutions of the VistaScan system in relation to the ability to diagnose proximal carious lesions and showed that there was no significant difference between the different resolution in agreement with similar previous studies (17-19). However, it is worth mentioning that the sensitivity was slightly higher than the specificity for all the resolutions and the accuracy was slightly higher for 25lp and 40lp resolutions compared to 20lp. Given this scenario, the use of 25lp would be recommended, since it showed better results than 20lp and equal results to 40lp, with the latter resolution being slower and generates images in heavier files. Furthermore, it is important to point out that the resolution of the Durr brand phosphor plates used in the study was 22lp, which could explain the better performance with scanning at 25lp (21). While the phosphor plates do not present an increased resolution to 40lp, the study of this parameter is not justified.

Li *et al*. (2008) (22) compared the 10lp and 20lp resolutions of the VistaScan system and 7.8lp and 12.5lp of the Digora system in the diagnosis of proximal carious lesions and concluded that an increased spatial resolution is not related to better detection of proximal caries, corroborating the findings of the present study. Wenzel *et al*. (2007) (18) compared the accuracy of caries diagnosis between different digital systems that provide more than one resolution and concluded that accuracy is little influenced by increased spatial resolution. Ferreira *et al*. (2019) (3) studied the influence of different resolutions of the VistaScan system for the detection of proximal caries and found that for the detection of enamel caries, the 10lp resolution was significantly higher than the others, in contrast to the results of the present study, although the 10lp resolution was not evaluated. The authors believe that the formation of noise in high-resolution images with smaller pixels makes it difficult to visualize small changes in enamel density and that high-resolution images, which are grainier, can mask or simulate the presence of small enamel lesions due to the lower level of X-ray dissipation between pixels.

Other studies have also evaluated the influence of resolution, albeit for different purposes; for example, Dantas *et al*. (2013) (23) analyzed the radiopacity of composites in conventional radiographs and digital images with different resolutions and showed that the high-resolution mode was the most efficient for radiographically differentiating dentin composites. Nejaim *et al*. (2016) (24) analyzed the influence of the number of lp on the accuracy of the detection of horizontal root fractures and concluded that the greater number of lp and, consequently, the higher image resolution seems to increase the accuracy of the diagnosis of horizontal fractures. In this last study, the authors did not use the same VistaScan system, thus differences between devices must be considered for their having obtained different results. On analyzing the spatial resolution relationship with external root resorption, Lacerda *et al*. (2020) (25) concluded that high resolution improved the diagnostic capacity and that it should be considered when performing digital radiographs.

To improve the diagnostic accuracy of caries lesions, different image processing techniques have been used, such as changes, in contrast, brightness, histogram equalization, noise reduction, and pseudocoloring (27). Some studies have demonstrated that the use of filters can improve the ability to diagnose carious lesions (10,18). Recently, Bardal *et al*. (2020) (28) compared two indirect digital systems regarding the detection of occlusal carious lesions in dentin in images with and without filters, and concluded that there were no significant differences between images treated with one of the tested filters and the original images. Likewise, Kositbowoenchai *et al*. (2004) (29) found no significant difference between images with or without filters in the diagnosis of occlusal caries.

In 2013, Belém *et al*. (30) evaluated the performance of digital radiographs with filters (negative, sharpened, and both) in the diagnosis of proximal caries lesions, using the Digora-Optime digital system. They concluded that images with a Sharpen filter showed better performance.

The present study evaluated the use of three different filters of the VistaScan digital system, Caries 1, Caries 2 and Fine. The Fine filter showed better sensitivity and accuracy values, but lower specificity than the Caries 1 filter. The Caries 2 filter showed lower specificity and accuracy values. The high specificity presented by the Caries 1 filter can prevent unnecessary procedures in the clinical setting. In a similar study, Haiter-Neto *et al*. (2009) (9) achieved similar results for the Fine filter in terms of sensitivity and accuracy and also described lower values for the Caries 2 filter. Thus, the use of the Fine filter can be recommended for the diagnosis of proximal carious lesions and the use of the Caries 2 filter should be avoided.

## Conclusions

According to our results, it is concluded that the time of demineralization interferes with the diagnostic capacity of carious lesions. Despite the lack of statistically significant differences in the variation of parameters, exposure time, resolution, and use of filters, it is recommended to use an exposure time of 0.2 s, to scan phosphor plates with 25lp and use Fine filters to achieve better diagnostic accuracy. It is also recommended to use the Caries 1 filter to improve specificity and prevent unnecessary procedures in the clinical setting.
